# Impact of ICU transfers on the mortality rate of patients with COVID-19: insights from comprehensive national database in France

**DOI:** 10.1186/s13613-021-00933-2

**Published:** 2021-10-26

**Authors:** Marc-Antoine Sanchez, Albert Vuagnat, Olivier Grimaud, Emmanuelle Leray, Jean-Marc Philippe, François-Xavier Lescure, Mathieu Boutonnet, Hélène Coignard, Agnès Ricard Hibon, Stephane Sanchez, Julien Pottecher

**Affiliations:** 1 Information Systems and Digital Department (DSIN), French Army Health Service, Saint Mandé-Bat 14, 69 avenue de Paris, 94165 Saint-Mandé, France; 2 Central Directorate of the Military Health Service (DCSSA), French Army Health Service, Paris, France; 3 Department for Research, Studies, Evaluation and Statistics (DREES), French Health and Social Affairs Ministry, Paris, France; 4 Univ Rennes, EHESP, REPERES(Recherche en pharmaco-épidémiologie et recours aux soins)-EA 7449, Rennes, France; 5 General Directorate for Health (DGS)-French Health and Social Affairs Ministry, Paris, France; 6 Tropical and Infectious Disease Services, Bichat AP HP, Paris, France; 7 INSERM 1137, Paris Diderot University, Paris, France; 8 Department of Anesthesiology and Intensive Care Unit, Percy Military Teaching Hospital, Clamart, France; 9 Emergency Medical Service, Lyon University Hospital, Lyon, France; 10 Emergency Medical Service, Pontoise Regional Hospital, Pontoise, France; 11 Department of Public Health and Performance, Troyes Hospital, Champagne Sud Hospital, Troyes, France; 12 Anaesthesiology, Critical Care and Perioperative Medicine, Strasbourg University Hospital-EA3072, FMTS, Strasbourg, France

## Abstract

**Background:**

The first wave of the COVID-19 pandemic confronted healthcare systems around the world with unprecedented organizational challenges, particularly regarding the availability of intensive care unit (ICU) beds. One strategy implemented in France to alleviate healthcare pressure during the first COVID-19 wave was inter-hospital transfers of selected ICU patients from overwhelmed areas towards less saturated ones. At the time, the impact of this transfer strategy on patient mortality was unknown. We aimed to compare in-hospital mortality rates among ICU patients with COVID-19 who were transferred to another healthcare facility and those who remained in the hospital where they were initially admitted to.

**Method:**

A prospective observational study was performed from 1 March to 21 June 2020. Data regarding hospitalized patients with COVID-19 were collected from the Ministry of Health-affiliated national SI-VIC registry. The primary endpoint was in-hospital mortality.

**Results:**

In total, 93,351 hospital admissions of COVID-19 patients were registered, of which 18,348 (19.6%) were ICU admissions. Transferred patients (*n* = 2228) had a lower mortality rate than their non-transferred counterparts (*n* = 15,303), and the risk decreased with increasing transfer distance (odds ratio (OR) 0.7, 95% CI: 0.6–0.9, *p* = 0.001 for transfers between 10 and 50 km, and OR 0.3, 95% CI: 0.2–0.4, *p* < 0.0001 for transfer distance > 200 km). Mortality decreased overall over the 3-month study period.

**Conclusions:**

Our study shows that the mortality rates were lower for patients with severe COVID-19 who were transferred between ICUs across regions, or internationally, during the first pandemic wave in France. However, the global mortality rate declined overall during the study. Transferring selected patients with COVID-19 from overwhelmed regions to areas with greater capacity may have improved patient access to ICU care, without compounding the short-term mortality risk of transferred patients.

**Supplementary Information:**

The online version contains supplementary material available at 10.1186/s13613-021-00933-2.

## Background

The first wave of the COVID-19 pandemic confronted healthcare systems around the world with unprecedented organizational challenges, particularly regarding availability of intensive care unit (ICU) beds. These challenges forced many hospitals to quickly adapt their capacity. In France, the first strategy implemented by the Ministry of Health (MOH) was to increase the number of ICU beds dedicated to patients with COVID-19, sometimes referred as ephemeral ICU beds, to avoid the peak saturation of hospitals that had been experienced in Italy [1, 2].

The scale and intensity of transfers of ICU patients with COVID-19 in 2020 was unprecedented. Inter-hospital transfers for ICU patients were rapidly undertaken during the first wave of the pandemic, moving patients from overcrowded ICUs towards less saturated ones [3–8]. The transfers were performed by hospital healthcare workers and administrative teams under the supervision of the regional health agencies (ARS) and the MOH. This was done to simultaneously improve not only the quantity, but also the quality of care since improved quality of care is expected from improving the nurse-to-patient ratio in less crowded areas and from increasing the global number of severely ill patients cared for in ICUs from impacted regions [9].

To match these goals, transfers had to be both safe (for transferred patients) and numerous (to offer a significant number of free ICU beds in the most impacted regions), which would imply a robust and consensual clinical criteria of transfer. Transfers were organized over both short distances within regions (intra-regional), over long distances between different regions (inter-regional), and between neighboring countries (international) and were also generally performed for one patient at a time. In practical terms, transfers were carried out with the support of French emergency medical services (SAMU) and the French Army Health Service (SSA) using dedicated (or customized) medical transport vehicles, trains, planes, boats, and helicopters, with physicians on board for the duration of the transfer [5].

Literature on the consequences of ICU transfer on patient mortality rates underlines two seemingly contradictory findings. On the one hand, transfers did not seem to increase mortality rates [10–15]. On the other, transfers were sometimes associated with worsened quality of care and/or clinical deterioration for transferred patients. The former was potentially due to pathway disruption and loss of information, the latter being associated with mobilizations, physical constraints that may alter both vital sign monitoring and provision of organ support difficult. The objective of this study was to describe the safety of the nationwide transfer strategy implemented in France during the first wave of the COVID-19 pandemic by comparing in-hospital mortality between ICU patients with COVID-19 who were transferred to another healthcare facility, and those who were not transferred in France within the same time frame.

## Methods

### Study design and participants

A prospective observational study was performed from 1 March to 21 June 2020. Patients were categorized as non-transferred if ICU care was conducted in the same unit to which they were initially admitted and categorized as transferred (either intra-regional, or inter-regional/international) if they were transferred to another ICU.

### Transfer criteria

The following clinical criteria were used to identify ICU patients with COVID-19 who were eligible for transfer: (i) stable hemodynamic and respiratory status; (ii) no requirement for extracorporeal oxygenation or prone positioning in the 48 h prior to transfer. Additionally, the following criteria had to be met to be eligible for plane and helicopter transfers: (i) inspired oxygen fraction less than 50%; (ii) positive expiratory pressure less than 10 cm H_2_O; (iii) noradrenaline infusion rate ≤ 0.15 μg.kg^−1^ min^−1^ and (iv) and body weight < 100 kg [16–18]. Consent for transfer was requested from the next of kin for all patients. When the first transfers were initiated, there were no publications validating formal criteria for the specific context of transferals of COVID-19 patient in ICUs during the first wave in France. Patients were chosen by experts (emergency and critical care physicians), considering their own experiences, estimated transfer duration and anticipated challenges. The chosen criteria were validated a posteriori*,* on December 17, 2020, by the National Emergency, Anesthesiology and Critical Care Medicine Societies and the French MOH and disseminated across healthcare services thereafter (See Additional file 1, “Critères de transférabilité inter-régionale d’un patient de soins critiques d’un établissement de santé à un autre”). Decision to transfer ICU patients with COVID-19 was taken on a local (intrahospital) basis, which was subsequently validated by territorial crisis management units and regional health agencies. Transfers were triggered when the possibility of opening extra ICU beds was exhausted and when the rate of contaminations and emergency room admissions was anticipated to result in ICU saturation within 48–72 h, precluding any supplemental ICU admission.

### Data source

Data on hospitalized patients with COVID-19 were collected from the national SI-VIC online health emergency registry. This database is designed to count victims and obtain real-time information about bed availability during large-scale health emergencies such as natural disasters, terrorist attacks or other exceptional healthcare situations, like the COVID-19 pandemic. Its data are exhaustive.

Since the start of the outbreak, all hospitals in France had recorded COVID-19 cases in the registry daily. COVID-19 cases were defined and standardized at the national level and had to be biologically confirmed with a positive RT-PCR test or highly suspected case based on a chest CT scan. A user’s guide to the SI-VIC registry was distributed to all hospitals to improve the quality of data collection. The registry included all hospital stays of patients with COVID-19 in acute units or ICU, in real-time, as well as with transfer data and hospital outcomes. All patients were identified with a unique anonymous national identifier, enabling all hospital stays to be identified and extracted for statistical analysis. All patients registered in SI-VIC and admitted to a French ICU for COVID-19 from 1 March to 21 June 2020 were included in the study. Follow-up was until July 14, 2020.

The following variables were extracted from the SI-VIC registry for each patient: (i) age; (ii) sex; (iii) origin (admitted from home, or from another hospital unit); iv) dates of admission to and discharged from the ICU or acute care unit; (v) destination after discharge from ICU or acute care unit (home, acute care unit, ICU, rehabilitation, death, still in ICU or acute care at the end of follow-up); (vi) length of acute care hospital stay (LOS) before ICU admission (days), LOS in ICU before transfer (days), LOS in ICU (days), total LOS (in acute unit and ICU, days); (vii) identification of the hospital; (viii) hospital type (university teaching hospital (CHU), general (non-academic) public hospital (CH), private not-for-profit hospital or private for-profit hospital (private)), and (ix) second admission to the ICU (binary: yes or no).

Patients were classified in one of three transfer categories: no transfer, intra-regional transfer, or inter-regional/international transfer. An additional binary variable (yes or no) was created to distinguish hospital stays of patients coming from regions considered to have “high incidence” at the time of hospitalization. In these four regions (from among a total of 13 regions in Metropolitan France), incidence and hospital admission rates rose sharply during the first semester of 2020, and ICU capacity was saturated > 100%, with daily incidence of new patients admitted to the ICU greater than five per million inhabitants. The Euclidian distance (km) was also calculated and recorded in the database to describe the distance between the sending and receiving hospitals, regardless of the means of transfer.

### Statistical analysis

Quantitative variables were described as mean ± standard deviation (SD) and qualitative variables as numbers and percentages. The Student’s t test was used to compare quantitative variables and the Chi-square test for qualitative variables. Mortality was plotted using the Kaplan–Meier method and curves were compared between groups using the log-rank test. For sensitivity analysis, we performed a Kaplan–Meier analysis in a restricted population, setting a start-time on the day of transfer, or on Day 5 of the ICU stay for non-transferred patients to take the immortality bias into account. This threshold of 5 days was chosen as it was the median LOS in ICU prior to transfer in the transferred population.

The primary endpoint was in-hospital acute care mortality. A modified Poisson regression model with robust error variance was computed to estimate adjusted risk ratios of patients and care characteristics on mortality. Due to the unavailability of numerous clinical variables (including severity scores), transferred group and non-transferred patient groups were not comparable. This, however, was not an objective of the study since our main goal was to challenge the safety of the nationwide transfer strategy. All available variables were included in the model. Adjusted risk ratios were computed with data of non-transferred patients present at Day 5 in ICU. Reported results were generated using SAS/STAT 14.3 (SAS Institute Inc., Cary, NC, USA). A mapping of transfer distances according to time was conducted to visually describe the chronological and spatial distribution of transfer types. Mapping was conducted with the PROC GMAP function of SAS.

### Ethics

The study design (prospective observational) was based on medical database that did not require patient consent according to French legislation [19]. The Ethics Committee of the French Society of Anesthesia and Intensive Care Medicine gave its approval for the study (IRB 00010254-2020-225).

## Results

**Fig. 1:**
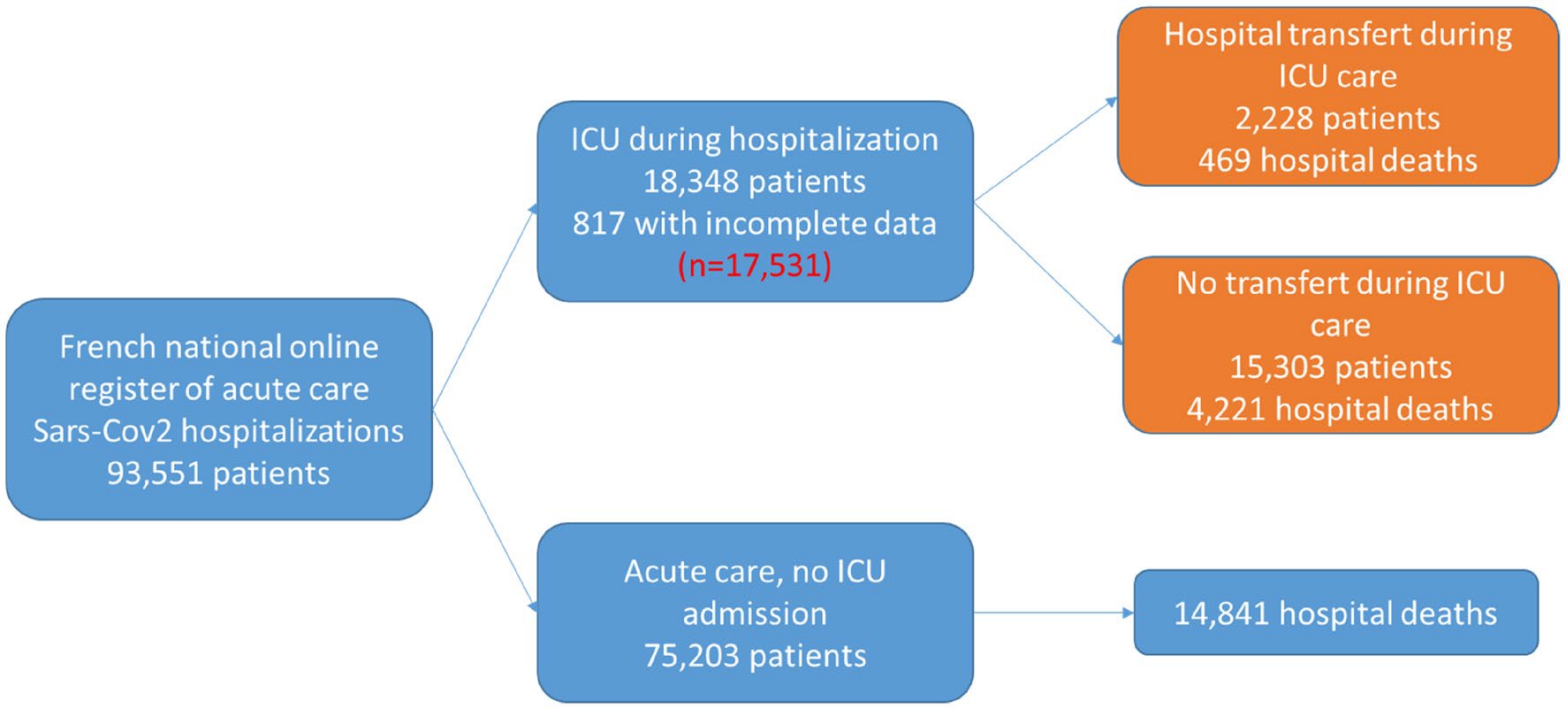
Flowchart of patients with COVID-19 from the SI-VIC national registry in France from 1 March to 21 June 2020. 328 patients were lost to follow-up during their ICU stay and 265 during acute care after ICU discharge because hospital staff did not update outcome information in the SI-VIC system; 216 patients had missing information (age and sex) and eight patients were admitted to hospital prior to study commencement (1 Feb 2020)

In total, 93,351 hospitalizations of patients with COVID-19 were recorded in the SI-VIC registry, of which 18,348 (19.6%) were ICU admissions. Complete data were available for 17,351 ICU admissions (95.6%), including 2228 transfers (12.7%). The flowchart of the study population is shown in Fig. 1. The mean age of patients admitted to the ICU was 63 ± 15.2 years; 69% were men. Transferred patients were younger than non-transferred patients (intra-regional patients 61.0 ± 13.2 years, inter-regional patients 61.7 ± 10.9, vs non-transferred patients 63.0 ± 15.5, *p* > 0.0001). Patient characteristics are detailed in Table 1.

**Table 1:** Characteristics of the study population according to transfer status for patients with COVID-19 hospitalized at an intensive care unit (ICU) in France between 1 March and 21 June 2020

	All patients (*n* = 17,351)	Non-transferred (*n* = 15,303)	Intra-regional transfer (*n* = 1623)	Inter-regional and international transfer (*n* = 605)	*P*-value
Age, years (mean, (SD))	62.8 (15.2)	63.0 (15.5)	61.0 (13.2)	61.7 (10.9)	< 0.0001
Men (%)	12,128 (69.2)	10,473 (68.4)	1,205 (74.3)	450 (74.4)	< .0001
Period of admission					
01–22 March 2020, *n* (%)	3699 (21.1)	3098 (20.4)	409 (25.2)	192 (31.7)	< 0.0001
23–29 March 2020, *n* (%)	4874 (27.8)	4058 (26.5)	535 (33.0)	281 (46.5)	
30 March to 5 April 2020, *n* (%)	3374 (19.3)	2915 (19.0)	369 (22.7)	90 (14.9)	
06–19 April 2020, *n* (%)	3160 (18.0)	2947 (19.2)	197 (12.1)	16 (2.6)	
20 April to 21 June 2020, *n* (%)	2424 (13.8)	2285 (14.9)	113 (7.0)	26 (4.3)	
Regions with high incidence, *n* (%)	11,975 (68.3)	10,203 (66.7)	1213 (74.7)	559 (92.4)	< 0.0001
Hospital admission					
University teaching hospital, *n* (%)	6411 (36.6)	5690 (37.2)	515 (31.7)	206 (34.0)	< 0.0001
General public hospital, *n* (%)	7813 (44.6)	6630 (43.3)	820 (50.5)	363 (60.0)	
Private hospital, *n* (%)	3307 (18.8)	2983 (19.5)	288 (17.8)	36 (6.0)	
Admission in ICU					
Directly from home, *n* (%)	11,190 (63.8)	9801 (64.1)	1031 (63.5)	358 (59.2)	0.0484
Following hospital admission, *n* (%)	6341 (36.2)	5502 (39.9)	592 (36.5)	247 (40.8)	
Length of stay in acute care prior ICU admission (days, (SD))	3.4 (5.2)	3.5 (5.4)	3.0 (3.9)	2.9 (3.5)	0.0486
Length of stay in ICU prior transfer, days (mean, (SD))	10.0 (12.9)	–	10.9 (14.1)	7.6 (8.3)	< 0.0001
Second admission in ICU, *n* (%)	654 (3.7)	535 (3.5)	96 (5.9)	23 (3.8)	< 0.0001
Length of stay in ICU, days (mean, (SD))	15.6 (16.6)	13.5 (14.6)	30.6 (21.8)	29.9 (19.3)	< 0.0001
Distance of hospital transfer during ICU					
Less than 10 km, *n* (%)	632	–	630 (38.8)	2 (0.3)	< 0.0001
10–50 km, *n* (%)	678	–	658 (40.5)	20 (3.3)	
50–200 km, *n* (%)	442	–	318 (19.6)	124 (20.5)	
200 km and more, *n* (%)	476	–	17 (1.1)	459 (75.9)	
Total length of stay in acute care, days (mean, (SD))	22.0 (18.7)	19.8 (17.0)	36.9 (22.7)	38.0 (20.1)	< 0.0001
Outcome at the end of follow-up					
Still in ICU	91 (0.5)	71 (0.5)	14 (0.9)	6 (1.0)	< 0.0001
Still in acute care	65 (0.4)	49 (0.3)	10 (0.6)	6 (1.0)	
Rehabilitation, convalescent or long-term care and nursing home	3272 (18.6)	2543 (16.6)	488 (30.1)	241 (39.8)	
Home	9465 (54.0)	8464 (55.3)	736 (43.3)	265 (43.8)	
Death	4638 (26.5)	4176 (27.3)	375 (23.1)	87 (14.4)	

The highest number of ICU admissions was during the week from 23 to 29 March 2020, and this week accounted for 46.5% of inter-regional transfers and 33% of intra-regional transfers during the study period. Most ICU admissions were in public hospitals (45%). Transferred patients were more often admitted to general (non-academic) public hospitals than non-transferred patients (50% among intra-regional transfers and 60% of inter-regional transfers).

Among all patients, 4% had two separate ICU admissions, while among transferred patients, 6% were admitted to the ICU twice. The mean LOS in acute care prior to ICU admission was 3.4 ± 5.2 days overall, 3.0 days (SD = 3.9) for intra-regional transferred patients and 2.9 days (SD = 3.5) for inter-regional transferred patients. The average LOS for all patients was 15.6 days (SD = 16.6) and 38 days (SD = 20.1) for inter-regional transferred patients. Non-transferred patients were more often discharged home (55.3%) than inter-regional (48.3%) and intra-regional (43.3%) transferred patients.

Regions performing inter-regional transfers abroad had a higher level of high COVID-19 incidence (92%) compared to all regions (68%). For the distance of transfers, 39% of intra-regional transfers were to a hospital within 10 km or less, while 76% of inter-regional transfers were over 200 km. The most diverse types of hospitals for transferred patients were observed between 30 March and 5 April 2020 (Additional file 2: Fig. S1).

Table 2 presents the mortality rates in the whole population and in transferred vs non-transferred patients by univariate and multivariate analysis. Mortality increased with age (OR = 26.7 in patients aged > 80 years compared to those aged 29 or less, (95% CI [16.7–42.7], *p* < 0.0001) and was higher in men (OR = 1.2, 95% CI [1.1–1.3], *p* < 0.0001) (Table 2). Mortality decreased over time with lower mortality after 20 April compared to the week from 23 to 29 March (OR = 0.8 95% CI [0.7–0.9], *p* < 0.0001) and from 1 to 23 March (OR = 0.5, 95% CI [0.4–0.5], *p* < 0.0001).

**Table 2:** Univariate and multivariate mortality models of patients with COVID-19 hospitalized at the intensive care unit (ICU) in France from 1 March to 21 June 2020

	Unadjusted model			Model with no population restriction			Model restricted on transferred patients and if not transferred still in ICU at Day 5		
	Unadjusted risk ratio	95% CI	*P-*value	Adjusted risk ratio	95% CI	*P*-value	Adjusted risk ratio	95% CI	*P*-value
Age									
≤ 29 years	1			1			1		
30–39 years	2.579	1.552–4.286	0.0003	2.450	1.541–3.896	0.0002	2.018	1.164–3.501	0.0124
40–49 years	2.739	1.702–4.408	< 0.0001	2.583	1.684–3.962	< 0.0001	1.873	1.142–3.075	0.0130
50–59 years	4.760	3.014–7.516	< 0.0001	4.466	2.961–6.736	< 0.0001	3.299	2.044–5.322	< 0.0001
60–69 years	7.374	4.688–11.598	< 0.0001	7.069	4.710–10.61	< 0.0001	5.057	3.152–8.112	< 0.0001
70–79 years	10.176	6.473–15.994	< 0.0001	9.889	6.593–14.832	< 0.0001	6.883	4.292–11.039	< 0.0001
≥ 80 years	12.837	8.148–20.225	< 0.0001	13.481	8.970–20.261	< 0.0001	8.966	5.574–14.42	< 0.0001
Sex (male)	1.105	1.036–1.177	0.0022	1.129	1.069–1.193	< 0.0001	1.177	1.103–1.255	< 0.0001
Hospitalization time period									
Up to 23 March 2020	1			1			1		
23–29 March 2020	0.813	0.751–0.879	< 0.0001	0.888	0.829–0.950	0.0006	0.842	0.780–0.910	< 0.0001
30 March to 05 April 2020	0.753	0.690–0.823	< 0.0001	0.818	0.763–0.878	< 0.0001	0.787	0.726–0.853	< 0.0001
06–19 April 2020	0.772	0.706–0.844	< 0.0001	0.765	0.712–0.822	< 0.0001	0.789	0.722–0.861	< 0.0001
20 April 2020 to 21 June 2020	0.610	0.549–0.678	< 0.0001	0.607	0.549–0.672	< 0.0001	0.580	0.514–0.655	< 0.0001
Region with high incidence									
GE, IDF, HDF, BFC vs others*	1.329	1.245–1.419	< 0.0001	1.504	1.418–1.595	< 0.0001	1.547	1.441–1.659	< 0.0001
Hospital type									
University teaching hospital	1			1			1		
Public hospital	1.038	0.973–1.106	0.2572	0.979	0.926–1.035	0.4614	0.940	1.003–0.881	0.0597
Private hospital	0.943	0.867–1.025	0.1670	0.825	0.767–0.887	< 0.0001	0.774	0.844–0.710	< 0.0001
Location before ICU admission (hospital vs home)	0.973	0.916–1.033	0.3661	0.954	0.906–1.005	0.0760	0.942	0.999–0.888	0.0468
ICU transfer									
Less than 10 km	0.922	0.787–1.080	0.3144	0.984	0.854–1.133	0.8221	1.005	0.870–1.159	0.9512
10–50 km	0.784	0.664–0.925	0.0039	0.791	0.679–0.922	0.0028	0.819	0.702–0.956	0.0114
50–200 km	0.846	0.695–1.029	0.0944	0.763	0.624–0.934	0.0088	0.788	0.642–0.967	0.0228
200 km and more	0.431	0.331–0.561	< 0.0001	0.382	0.291–0.500	< 0.0001	0.392	0.298–0.514	< 0.0001

Being hospitalized in a region with high COVID-19 incidence was associated with an increased risk of death (OR = 1.8, 95% CI [1.7–2.0], *p* < 0.0001). Being admitted directly to the ICU from home was not associated with higher mortality (OR = 0.9, 95% CI [0.9–1.0], *p* = − 0.0537). The risk of mortality was also less than half during a second ICU stay (OR = 0.5; 95% CI [0.4–0.6], *p* < 0.0001).

**Fig. 2:**
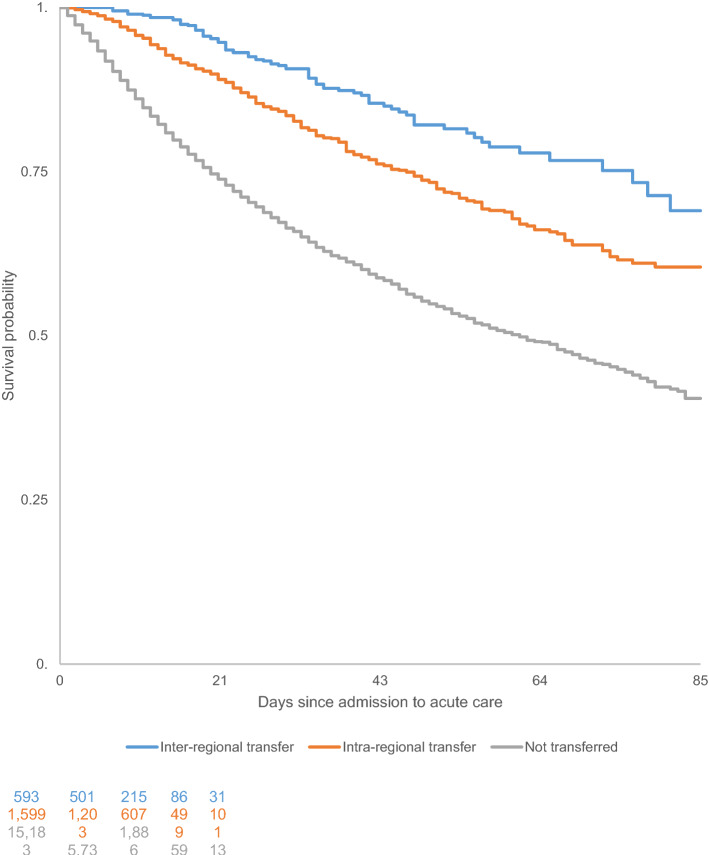
Kaplan–Meier survival curves for ICU patients with COVID-19 in France who were transferred versus those who were not transferred from 1 March to 21 June 2020

**Fig. 3:**
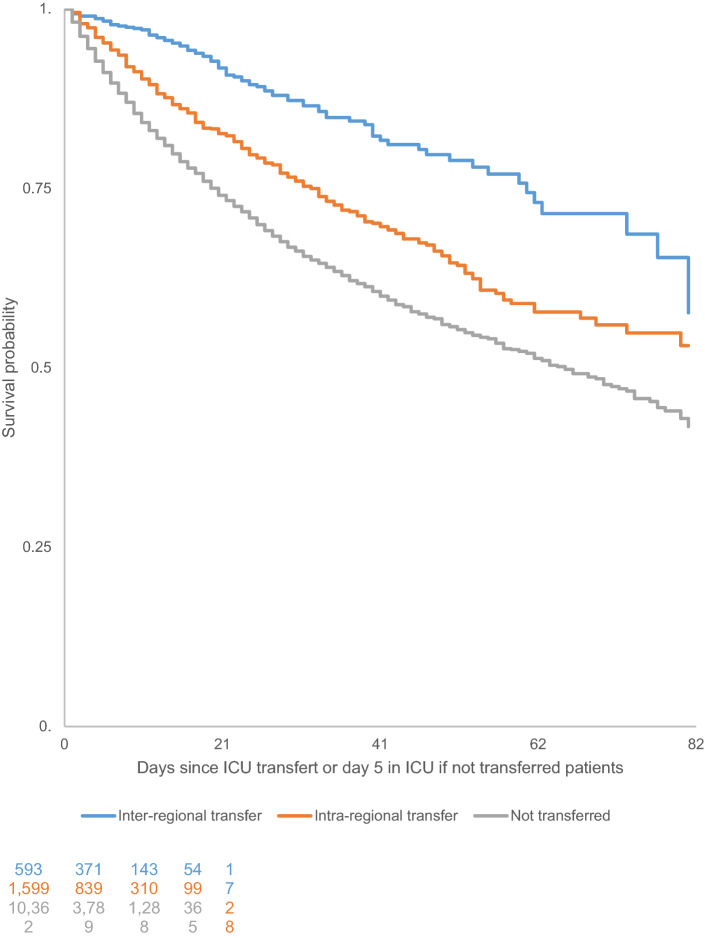
Kaplan–Meier survival curves for COVID-19 patients in the ICU in France who were transferred versus those who were not transferred from 1 March to 21 June 2020 among patients hospitalized for 5 days or longer

Mortality was lower among transferred patients compared to non-transferred patients, as shown by the Kaplan–Meier survival curves and log-rank test (*p* < 0.0001) (Figs. 2, 3). Transfers were associated with a lower mortality risk, and the risk decreased with increasing transfer distance (Table 2). Sensitivity analysis performed on transferred patients *vs.* non-transferred patients who were still in the ICU on Day 5 provided similar findings (Table 3).

**Table 3:** Univariate and multivariate mortality models of patients with COVID-19 still hospitalized on Day 5 in intensive care unit (ICU) from 1 March to 21 June 2020

	Risk ratio	95% CI	*P*-value	Risk ratio	95% CI	*P*-value
Age						
≤ 29 years	1			1		
30–39 years	2.145	1.184–3.885	0.0118	2.018	1.164–3.501	0.0124
40–49 years	1.957	1.121–3.415	0.0181	1.873	1.142–3.075	0.0130
50–59 years	3.473	2.041–5.912	< 0.0001	3.299	2.044–5.322	< 0.0001
60–69 years	5.179	3.056–8.776	< 0.0001	5.057	3.152–8.112	< 0.0001
70–79 years	6.935	4.095–11.747	< 0.0001	6.883	4.292–11.039	< 0.0001
≥ 80 years	8.314	4.885–14.150	< 0.0001	8.966	5.574–14.42	< 0.0001
Sex (male vs female)	1.147	1.060–1.240	0.0006	1.177	1.103–1.255	< 0.0001
Hospitalization time period						
Up to 23 March 2020						
23–29 March 2020	0.777	0.710–0.851	< 0.0001	0.842	0.780–0.910	< 0.0001
30 March to 05 April 2020	0.739	0.666–0.819	< 0.0001	0.787	0.726–0.853	< 0.0001
06–19 April 2020	0.789	0.709–0.878	< 0.0001	0.789	0.722–0.861	< 0.0001
20 April 2020 to 21 June 2020	0.578	0.504–0.663	< 0.0001	0.580	0.514–0.655	< 0.0001
Region with high incidence (vs others^a^)	1.347	1.243–1.459	< 0.0001	1.547	1.441–1.659	< 0.0001
Hospital type						
Public hospital vs university hospital	0.972	0.901–1.048	0.4584	0.940	0.881–1.003	0.0597
Private hospital vs university hospital	0.868	0.785–0.960	0.0059	0.774	0.710–0.844	< 0.0001
Location before ICU admission (Hospital vs Home)	0.958	0.892–1.029	0.2442	0.942	0.888–0.999	0.0468
ICU transfer						
Less than 10 km	0.941	0.802–1.105	0.4588	1.005	0.870–1.159	0.9512
10–50 km	0.800	0.677–0.946	0.0089	0.819	0.702–0.956	0.0114
50–200 km	0.864	0.709–1.052	0.1454	0.788	0.642–0.967	0.0228
200 km and more	0.440	0.338–0.573	< 0.0001	0.392	0.298–0.514	< 0.0001

^a^Grand Est, Ile-De-France, Hauts-de-France, Burgundy, Franche-Comté

## Discussion

Our study demonstrated that intra-regional, inter-regional or international ICU transfers of ICU COVID-19 patients was associated with a lower risk of death during the first epidemic wave in France. This suggests that the strategy of spreading the burden of care across a country according to available capacity was not prejudicial to the patients’ outcome, even though the two groups (transferred vs*.* non-transferred) may not have been comparable at baseline. Each transfer indication was validated by trained teams with an agreement in place between regional stakeholders, thanks to successful national coordination. The efficiency of this strategy must be considered, keeping in mind the limited resources available at that time. Considering recent US experience showing that strain on critical care capacity was associated with increased COVID-19 ICU mortality [20], these results appear clinically relevant and deserve further exploration.

Average mortality (26.5%) was lower in the group of transferred patients, especially for inter-regional transfers (14.4%) regardless of the LOS. The risk of death in transferred patients also decreased with increasing distance between the sending and receiving ICU. These results likely reflect the appropriate selection of patients for transfer, namely those deemed clinically stable with better prognosis and who could best withstand long distance transfer. The organization of transfers can have a negative impact on the quality and safety of care and this may have been a risk during handovers between teams and during transfer itself [21, 22].

Differences were found between transferred and non-transferred patients where more patients with inter-regional transfer were men, younger, admitted to general (non-academic) public hospitals, and more frequently referred to rehabilitation services compared to the other groups. Overall, the choice of intra- or inter-regional represented two different approaches. The approach depended on bed availabilities and type of transfer concerning a different patient population. Intra-regional transfers were performed for three main reasons: (i) increase in the level of care; (ii) need to free beds in some specialized (cardiosurgical, trauma, transplantation) ICUs or (iii) expected long weaning from mechanical ventilation. The proportion of each is currently unknown. Most inter-regional transfers were performed to make ICU beds available in regional hospitals most affected by the outbreak.

A period effect on mortality was also observed in this study. Mortality was higher in the first few weeks of the epidemic surge and declined in the later weeks of the study period. This decrease in mortality may be due to the learning effect of practitioners at individual and team levels regarding treatment of the new disease, through experience and availability of new scientific knowledge and guidelines [23, 24]. Comparisons between mortality patterns during the second wave are warranted to confirm our hypothesis. This qualitative improvement may also have been made possible by the quantitative adaptation of resources because the health reserve mobilized additional medical and paramedical human resources. This measure helped to guarantee an optimal level of care since the ratio of patients to caregivers is known to directly influence the quality of care [25]. The significance of this additional measure was further highlighted when further mobilization became necessary to cope with the second wave.

In France, half of all transfers occurred around ten days before the peak of the epidemic’s first wave. This occurrence likely helped to alleviate the pressure on ICUs by increasing local capacity for admission of new patients, especially in tension regions with high incidence. Therefore, transferring patients when it was technically possible, helped to achieve a more even distribution of the epidemic burden across the country, which may have improved quality of care. This approach was made possible thanks to a coordinated definition of rules, criteria, processes and management of transfers without increasing the strain on the receiving region [26]. Due to the dynamics of the COVID-19 outbreak, regions under tension needing to transfer patients abroad at one point in time may have later been relieved and able to accept patients transferred from other areas. It is therefore worth stating that monitoring rules and coordination of transfers at a national level on a regular basis and considering epidemiological and organizational criteria in addition to clinical criteria is necessary. Moreover, using projections of bed occupancy rates by region could be a relevant tool to guide decision-making for transfers [27].

LOS should also be considered when estimating bed occupancy rates, as the LOS was almost twice as long in transferred patients compared to non-transferred patients. Nevertheless, LOS in acute care prior to ICU admission was almost the same in all groups. The transfer itself could partially explain the increase in the LOS due to the time required to prepare the patient for transfer, the duration of the transfer, and time for the new team to get acquainted with the patient. However, this assertion remains to be verified. Clinical factors such as disease severity and occurrence of a complication during a stay are additional factors that could explain the increased LOS in ICU among transferred patients [28]. Further studies are warranted to investigate the factors driving the LOS. An increased LOS in the ICU exposes the patient to a higher risk of healthcare-associated infection and this needs further exploration [29]. Conversely, patients transferred intra-regionally were readmitted to the ICU more often than other patients which may be explained by premature discharge, where the care was not optimal, or was subsequently worsening (associated with co-morbidities or complications) [30].

The main strengths of this study resided in the quality of the data used. Data were collected from the only national database containing all prospectively recorded COVID-19 cases in France with standardized case definitions. Additionally, all transfers were recorded in the database, limiting the risk of selection bias in comparing the two groups. Patient and outbreak characteristics in this study are similar to those reported in the literature and epidemiological reports from Public Health France, particularly concerning the peak surge in ICU-bed occupancy on April 8, 2020 [31, 32]. The main limitation of our analysis was the lack of clinical, socio-demographic and management data for the study population. Collection of this information is currently ongoing as part of the TRANSCOV project (ClinicalTrials.gov Identifier: NCT04930120) and should enable more detailed comparison of transferred *vs.* non-transferred patients and enhance our understanding of the variations of medium-term outcomes.

## Conclusion

Our study showed that inter-regional and international collaborations for the transfer of ICU patients with COVID-19 by qualified medical services were an effective and safe strategy in a large-scale outbreak. This strategy made it possible to alleviate the strain imposed on high-incidence ICUs. Transfers between ICUs did not adversely affect short-term mortality thanks to the rigorous selection process of patients eligible for transfer. Thus, we emphasize that transfers were useful because they allowed the preservation of a surge capacity in most affected regions while not exposing transferred patients to undue harm. Since it is incorporated in such a comprehensive strategy, transfers have allowed the global system efficiency to maintain itself. This strategy should be encouraged in countries where medical transfer capacities and skilled healthcare workers are available, as it can relieve the pressure on ICUs during a surge in demand. At a global level and based on acquired experience, guidelines for patient transfers in case of upcoming health emergencies should be considered in the future.

## Supplementary Information

**Additional file 1**: Criteria for inter-regional transferability of a critical care patient between two hospitals.

**Additional file 2**: Map of intensive care unit (ICU)-transfers of hospitalized patients with COVID-19 patients in France from 1 March to 21 June 2020.

## Data Availability

Data are available upon request to the first author.
