# Supplementary material for: Impact of ICU transfers on the mortality rate of patients with COVID-19: insights from comprehensive national database in France
Source: Ann Intensive Care. 2021 Oct 26;11:151. doi: 10.1186/s13613-021-00933-2 (PMC8546754; doi:10.1186/s13613-021-00933-2)
Supplement: Supplementary file 1 — Additional file 1: Criteria for inter-regional transferability of a critical care patient between two hospitals. [file 13613_2021_933_MOESM1_ESM.docx]

**Criteria for inter-regional transferability of a critical care patient between two hospitals**

Real time or future saturation of critical care beds in one region may lead to a decision to transfer patients to the critical care unit of a hospital in another region. These transfers are not carried out for the benefit of the patients transferred but for patients who will require critical care beds in the near future.

These transfers can only be considered if:

- they are safe for the patient, including if they are validated on the criteria for transportability.

- the transferred patient's care project is defined and shared with the receiving team.

- they are part of a process of making a critical care bed available, for example, so that the expected length of stay in intensive care after transfer is greater than two days.

Criteria are listed in the table below.

| **Criteria** | **Attention point** | **Yes** | **No** |
| --- | --- | --- | --- |
| **Transportability** Major Criteria | Agreement of the family |  |  |
|  | Length of stay in critical care unit> 24 hours |  |  |
|  | Weight< 100kg |  |  |
|  | Noradrenaline< 1mg/h |  |  |
|  | No extra-renal epuration |  |  |
|  | Positive expiratory pressure P< 15cm H_2_O |  |  |
|  | FiO_2_≤ 0.6 |  |  |
|  | Absence of prone position for at least 12 hours |  |  |
|  | No Multi Resistant Bacteria |  |  |
|  | No Extracorporeal membrane oxygenation |  |  |
|  | Closed suction system in place |  |  |
|  | Number of Electric Syringe Pushers≤ 4 |  |  |
| **Patient's project of care:** Major criteria | Family able to receive information by telephone and/or to stay secondarily with patient in the context of the measures taken to this end. |  |  |
|  | High survival probability |  |  |
|  | No formalised Therapeutic Activity Limitation |  |  |
|  | Probability of mechanical ventilation> 48 hours and weaning not started. |  |  |
|  | No early prolonged weaning |  |  |
| **Regional Optimization:** Minor criteria | Probability of ICU stay> 48 hours |  |  |

ICU; Intensive Care Unit
