# Supplementary material for: Impact of ICU transfers on the mortality rate of patients with COVID-19: insights from comprehensive national database in France
Source: Ann Intensive Care. 2021 Oct 26;11:151. doi: 10.1186/s13613-021-00933-2 (PMC8546754; doi:10.1186/s13613-021-00933-2)

**Supplementary Figure 1.** Map of intensive care unit (ICU)-transfers of hospitalized patients with COVID-19 patients in France from 1 March to 21 June 2020


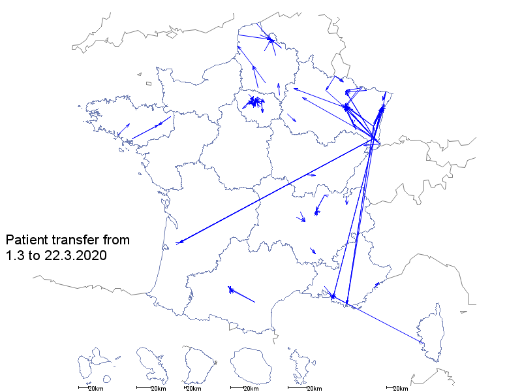

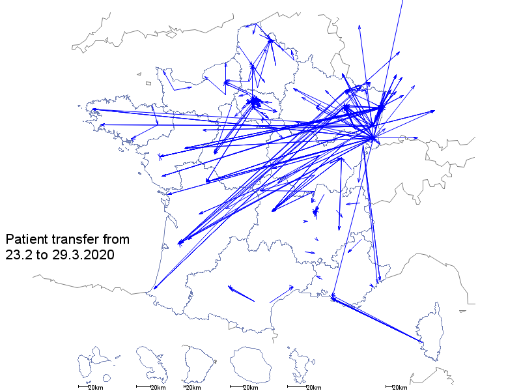

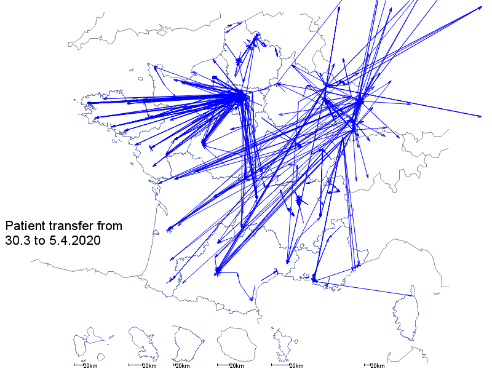


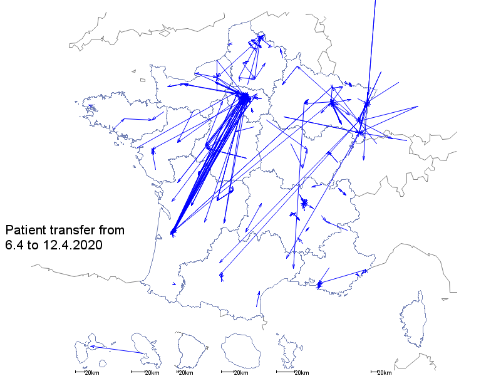

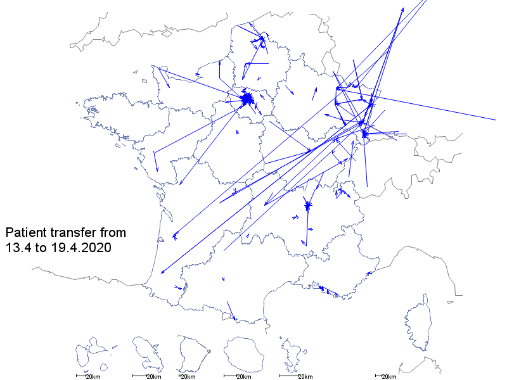

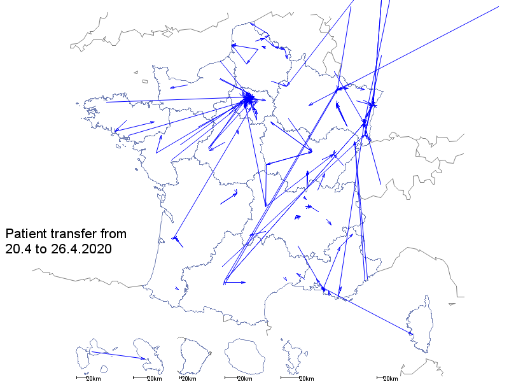


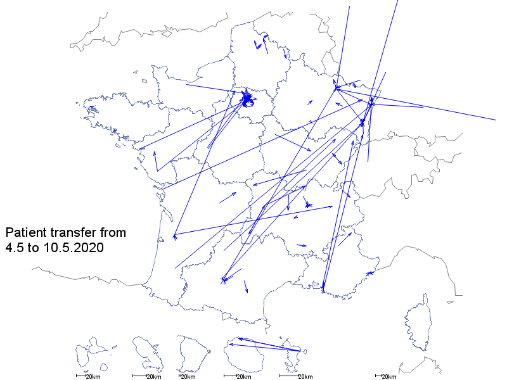

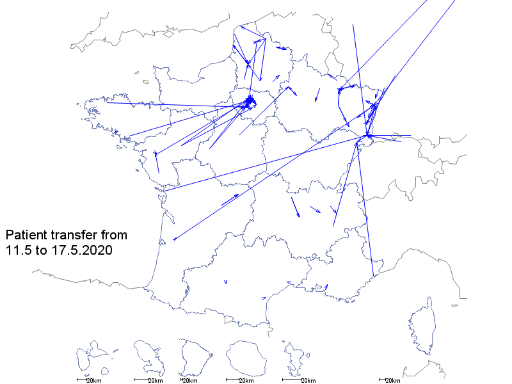

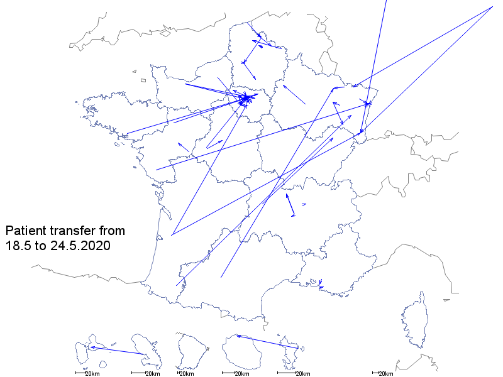


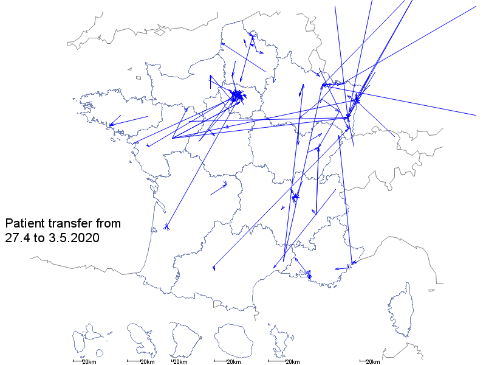

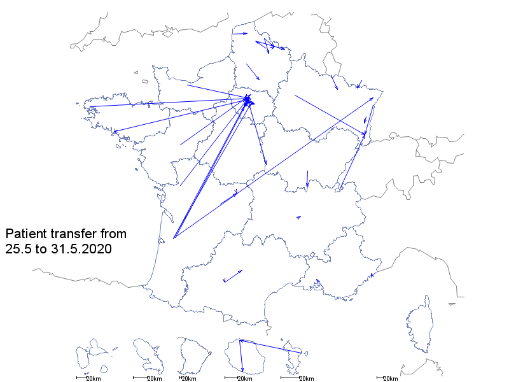

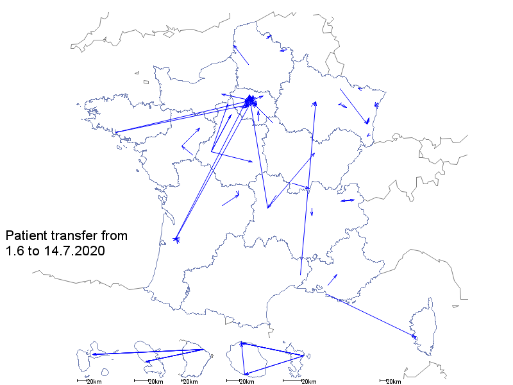

Supplement: Supplementary file 2 — Additional file 2: Map of intensive care unit (ICU)-transfers of hospitalized patients with COVID-19 patients in France from 1 March to 21 June 2020. [file 13613_2021_933_MOESM2_ESM.docx]
